# Parental Selection of Hybrid Breeding Based On Maternal and Paternal Inheritance of Traits in Rapeseed (*Brassica napus* L.)

**DOI:** 10.1371/journal.pone.0103165

**Published:** 2014-07-25

**Authors:** Nailin Xing, Chuchuan Fan, Yongming Zhou

**Affiliations:** National Key Laboratory of Crop Genetic Improvement, Huazhong Agricultural University, Wuhan, China; Wuhan University, China

## Abstract

Parental selection is crucial for hybrid breeding, but the methods available for such a selection are not very effective. In this study, a 6×6 incomplete diallel cross was designed using 12 rapeseed germplasms, and a total of 36 hybrids together with their parental lines were planted in 4 environments. Four yield-related traits and seed oil content (OC) were evaluated. Genetic distance (GD) was estimated with 359 simple sequence repeats (SSRs) markers. Heterosis levels, general combining ability (GCA) and specific combining ability (SCA) were evaluated. GD was found to have a significant correlation with better-parent heterosis (BPH) of thousand seed weight (TSW), SCA of seeds per silique (SS), TSW, and seed yield per plant (SY), while SCA showed a statistically significant correlation with heterosis levels of all traits at 1% significance level. Statistically significant correlations were also observed between GCA of maternal or paternal parents and heterosis levels of different traits except for SS. Interestingly, maternal (TSW, SS, and OC) and paternal (siliques per plant (SP) and SY) inheritance of traits was detected using contribution ratio of maternal and paternal GCA variance as well as correlations between GCA and heterosis levels. Phenotype and heterosis levels of all the traits except TSW of hybrids were significantly correlated with the average performance of parents. The correlations between SS and SP, SP and OC, and SY and OC were statistically significant in hybrids but not in parents. Potential applications of parental selection in hybrid breeding were discussed.

## Introduction

Plant breeding currently faces the daunting task of meeting the challenges posed by global climate change and a growing need for food supply and bioenergy. Heterosis has proven to be an efficient way to improve yield and environmental adaptability, and has been used in breeding of many crops [1]–[3]. Rapeseed (*Brassica napus*), an important oilseed crop worldwide, is one of the most successful crops in application of heterosis [4]–[7]. So far, the vast majority of the hybrid cultivars has been developed through extensive selection of F_1_ hybrid combinations, thus involving high cost and a lengthy turn-around period. Therefore, it is necessary to explore efficient methods that could predict hybrid performance in the parental generation.

In the past two decades, a number of studies have been reported that investigated methods for hybrid performance prediction in rapeseed. Riaz et al. and Tan et al. found a significant correlation between genetic distance (GD) and yield heterosis through molecular markers [8]–[9]. Ali et al. found significant correlations between GD and yield-component traits, but no significant correlation was identified for the number of branches per plant [10]. However, Yu et al. found significant correlations between some agronomic traits, but not for seed yield [11]. Diers et al. reported that GD was significantly correlated only with heterosis of seed yield in inbred diallel combinations, but not with cultivar diallel [12]. Qian et al. found no significant correlations between heterosis and GD, but significant correlations between general combining abilities (GCA) and hybrid performance [13]. Similarly, Teklewold and Becker, and Devi and Singh both found significant correlations between combining abilities and hybrid performance [14]–[15]. However, no effective method of parental selection has been proposed through GD and combining abilities in hybrid breeding.

A thorough understanding of the inheritance mode of traits is essential to establish an effective method for parental selection. Inheritances of traits have been analyzed widely in bi-parent populations and diallel crosses in rapeseed, and additive, dominance and epistasis effects have been identified for a wide array of traits [16]–[20]. In addition, maternal and paternal inheritances of traits have been studied using reciprocal crosses in many crops [21]–[23]. Maternal and paternal effects of heterosis have been identified only through analyzing GCA variance contributed by different parents [24]. However, no report is available about different correlations between traits in parents and hybrids, together with the correlations between heterosis levels and GCA of different parents.

The objectives of the current study are to investigate: (1) the relationships between GD, combining abilities, and heterosis levels, (2) maternal and paternal contribution to hybrid performances through analysis of GCA variance and correlations between heterosis levels and GCA of different parents, and (3) correlations between traits with parents as homozygous genotypes and hybrids as heterozygous genotypes. Our results provide novel information for parental selection in hybrid breeding of rapeseed.

## Materials and Methods

### Plant materials and field experiments

The experimental materials used in this study comprised 12 semi-winter rapeseed germplasms including 5 cultivars and 7 inbred lines whose characteristics was described in Table S1. With the parental lines listed in Table S1, a total of 36 hybrids were developed using a 6×6 incomplete diallel cross design (Table 1).

**Table 1:** Genetic distance between parental lines used for 6×6 incomplete diallel crosses.

Parental lines	P1	P2	P3	P4	P5	P6
P7	0.36	0.38	0.21	0.72	0.44	0.61
P8	0.21	0.40	0.40	0.66	0.43	0.61
P9	0.41	0.50	0.58	0.33	0.53	0.44
P10	0.37	0.41	0.44	0.53	0.40	0.46
P11	0.46	0.45	0.54	0.57	0.45	0.46
P12	0.47	0.49	0.56	0.50	0.52	0.46

P1 to P6: maternal parents; P7 to P12: paternal parents.

P1, P2, P3, P7, and P8: cultivars (P1: ZS5; P2: ZS7; P3: ZS10; P7: ZS9; P8: HS5). P4 to P6, and P9 to P12: inbred lines.

See also Table S1 for detailed characteristics of the parental lines.

The hybrids along with the parents were grown in 4 natural environments at 3 different locations. They were planted in a winter rapeseed crop area at the experimental farm of Huazhong Agricultural University, Wuhan, China in the season of 2009–2010 and 2010–2011, and Huanggang Academy of Agricultural Sciences, Huanggang, China in the season of 2010–2011 [25], and in a spring rapeseed crop area in Hezheng, Gansu, China in the season of 2010. The field experiments of Huanggang were granted permission by the administrative board of the Huanggang Academy of Agricultural Sciences. The experiment location of Gansu was the experiment base of Huazhong Agricultural University, and no specific permission was required for the field trial. All parents and hybrids were used for all the environments and growing seasons and were grown for the evaluation of phenotype traits at the mature plant stage. All the field trials in this study did not involve endangered or protected species.

All the field experiments followed a randomized block design with 3 replications, and each replication comprised parents and hybrids that were planted in 4 rows with 48 plants for each plot. The crop was cultivated with standard agronomic practices. Ten mature plants in the middle of each plot were randomly selected for trait evaluation, and the mean value of each trait was used for analysis. Seed yield per plant (SY), seeds per silique (SS), siliques per plant (SP), thousand seed weight (TSW), and oil content in seeds (OC) were evaluated as described by Shi et al. [19].

### Genetic diversity

Genomic DNA was isolated following the standard procedure [26]. A total of 359 simple sequence repeats (SSRs) including all the markers mapped on the linkage groups constructed by Fan et al. [27] were used to analyze the genetic diversity of parents. Genetic diversity was estimated on the basis of polymorphism generated by SSR primers. Amplification profiles of test genotypes were compared with each other and bands of DNA fragments were scored as ‘1’ for present and ‘0’ for absent. The genetic distance was computed using UPGMA method by NTSYS_PC_ Version 2.10e [28].

### Statistical analysis

The mid-parent heterosis (MPH), better-parent heterosis (BPH), general combining ability (GCA), specific combining ability (SCA) and correlation coefficients were calculated using SAS v9.3 software (SAS Institute, Cary, North Carolina, USA) using the GLM procedure [29]–[30].

The MPH was calculated as: MPH (%)  =  (F_1_ – MP)/MP ×100, BPH (%)  =  (F_1_ – BP)/BP ×100. where MP is the mean of the two parents, and BP the value of the better parent.

For combining ability analyses, the GCA for parents and SCA for hybrids were calculated using the following formula, respectively:
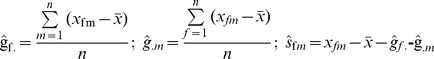



Here, 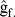, 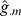, and 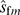 represent the GCA of female and male parent, and SCA of the hybrid, respectively; 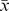 represents the mean value of all the hybrids; 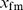 represents the value of the hybrid produced with the fth female parent and the mth male parent; n represent the number of female or male parents. The variance of additive effect (A), dominant effect (D), additive by additive effect (AA), and their interaction with environment (AE, DE, and AAE) were estimated using QGA Station Version 1.0 software [31]. The broad sense of heritability (h^2^b), narrow sense heritability (h^2^n), variance of GCA, variance of SCA, and ANOVA analysis were conducted using DPS software [32]. Heritability was calculated using the following formula:
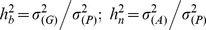



Here, 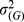 is the genotypic variance, 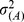 is the additive variance, and 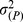 is the phenotypic variance [24]. MPH and BPH of hybrids, and GD of the parents between the hybrids, as well as GCA and SCA of the parents and hybrids were correlated with each other to assess the relationship between these genetic parameters and parental diversity.

The maternal and paternal inheritances were analyzed by calculating the correlation coefficients between the hybrids and their parents and comparing the contribution of female and male genotypic variance to total genotypic variance for each trait [24]. The correlation coefficients between the traits investigated were computed through phenotypic performance of parents and hybrids, respectively. The correlation coefficients between the hybrids and their maternal or paternal lines were computed through phenotypic performance of each trait.

## Results

### Estimation of variance and covariance components

Variances of genetic effect (G), environment effect (E), and G×E were statistically significant for all traits in parents, hybrids, and all the plant materials including parents and hybrids at the 1% significance level, except for the environment effect of SY in hybrids and SP in all the plant materials (Table 2). In addition, the additive, dominant and additive by additive interaction effects were statistically significant for all traits at the 1% significance level, except for the additive effect of SY, dominant effect of SS and AA of SP and TSW (Table 3). For the genetic and environment interaction effect, AE of TSW and OC, and AAE of SY, SS, and SP were not significant. For SP, the dominant effect was greater than the additive effect. On the contrary, the additive effect was higher than the dominant effect for TSW and OC. These results indicated that SY of hybrid was mainly controlled by the dominant and epistasis effects, SS was mainly controlled by the additive and epistasis effects, SP was mainly controlled by the dominant effect, and TSW and OC were mainly controlled by the additive effect. Furthermore, the SY of hybrids had stronger environment adaptability than parents did, which was consistent with prior research [33].

**Table 2:** Double factors variance analysis of traits.

		DF	SY	SS	SP	TSW	OC
Parent	G	11	191.54**	110.15**	59614.20**	2.36**	34.29**
	E	3	104.47**	24.01**	32519.32**	0.78**	71.83**
	G×E	33	29.79**	65.05**	8293.86**	0.75**	14.77**
Hybrid	G	35	300.44**	98.26**	70730.35**	2.86**	30.89**
	E	3	5.68	126.44**	6482.04**	2.90**	28.71**
	G×E	105	21.50**	12.80**	7499.03**	0.22**	5.65**
Total	G	47	326.60**	107.03**	71818.80**	2.76**	48.46**
	E	3	34.52**	61.92**	923.54	1.57**	66.22**
	G×E	141	24.59**	26.64**	8335.66**	0.39**	10.17**

G: genotype; E: environment; G×E: interaction of genotype and environment; DF: degree of freedom.

SS: seeds per silique; SP: siliques per plant; SY: seed yield per plant; TSW: thousand seed weight; OC: oil content in seeds; MPH: middle parent heterosis; BPH: better parent heterosis. The same abbreviations were used below.

** Significant at P = 0.01

**Table 3:** Variance analysis of traits (By QGA station).

	SY	SS	SP	TSW	OC
A	0	1.76**	592.14**	0.24**	1.15**
D	0.45**	0	961.36**	0.13**	0.29**
AA	6.46**	2.59**	0	0	0.38**
AE	2.89**	3.11**	1423.52**	0	0
DE	8.66**	3.65**	2695.86**	0.02**	0.23**
AAE	0	0	0	0.03**	0.56**

A: additive effect; D: dominant effect; AA: interaction of additive and additive effect; AE: interaction between additive and environment effect; DE: interaction between dominant and environment effect; AAE: interaction of epistasis of additive and additive effect and environment effect.

** Significant at P = 0.01.

### Heterosis performance of hybrids

The MPH ranged from −12.88% of SP for P6×P8 to 96.17% of SY for P2×P11, and SY showed the highest average MPH (45.41%) among the 5 traits (Table 4). The BPH ranged from −22.78% of SS for P5×P8 to 64.64% of SY for P2×P11, and SY also had the highest average BPH (26.07%) among the 5 traits. The average MPH and BPH showed a positive direction for all the traits, except for the average BPH of SS and TSW. This result was consistent with prior research [9].

**Table 4:** Heterosis level of incomplete diallel crosses.

Traits	MPH (%)		BPH (%)	
	Mean ±SD	Range	Mean ±SD	Range
SY	45.41±0.21	8.10∼96.17	26.07±0.17	−3.35∼64.64
SS	9.79±0.08	−11.29∼22.59	−2.07±0.07	−22.78∼10.51
SP	18.75±0.14	−12.88∼45.76	7.53±0.15	−19.98∼36.93
TSW	6.39±0.07	−9.00∼21.94	−2.66±0.09	−23.22∼16.71
OC	3.95±0.02	0.06∼7.23	0.63±0.02	−5.02∼3.88

### Heritability, combining abilities and GD

For the 5 traits, h^2^b and h^2^n ranged from 68.27% to 93.05%, and 18.61% to 74.88% respectively (Table 5). TSW showed the highest heritability, whether h^2^b or h^2^n, followed by SS, while the h^2^n of SY was only 18.61%.

**Table 5:** Genetic parameters of heritability and combining ability.

Traits	V_G_ (%)	V_S_ (%)	V_gf_ (%)	V_gm_ (%)	V_gfm_ (%)	h^2^_b_ (%)	h^2^_n_ (%)
SY	25.71	74.30	10.32	13.70	75.98	68.77	18.61
SS	68.56	31.44	42.96	26.47	30.58	89.67	61.66
SP	66.13	33.87	29.97	39.26	30.78	75.12	40.06
TSW	80.47	19.53	69.52	11.30	19.18	93.05	74.88
OC	70.39	29.61	35.80	34.83	29.37	68.27	48.18

V_G_: variance of general combining ability (GCA); V_S_: variance of specific combining ability (SCA); V_gf_: contribution ratio of the female GCA variance to the total variance; V_gm_: contribution ratio of the male GCA variance to the total variance; V_gfm_: contribution ratio of the SCA variance to the total variance; h^2^_b_: broad sense heritability; h^2^_n_: narrow sense heritability.

The variance of GCA was higher than that of SCA for all traits except for SY. Additionally, TSW showed the highest GCA variance of 80.47% among the 5 traits while SY showed the lowest GCA variance of 25.71%. Furthermore, maternal genotypic variance was found to contribute more to total genotypic variance than paternal one for SS, TSW, and OC, but less for SY and SP. The maternal and paternal contributions to TSW showed the greatest difference among the 5 traits (Table 5).

A total of 359 SSR markers were used to estimate the genetic diversity of the parents. The number of polymorphic alleles generated by the markers ranged from 1 to 11 with an average of 3. The genetic distances between both parents of the hybrids ranged from 0.21 for P3×P7 and P1×P8 to 0.72 for P4×P7 (Table 1).

### Relationships of GD, combining abilities, and heterosis levels

No significant relationship was found between GD and MPH for all 5 traits (Table 6), which was consistent with prior reports [1], [13]. However, a negative and statistically significant correlation was detected between GD and BPH of TSW at the 1% significance level. In addition, statistically significant correlations were detected between GD and SCA of SS, TSW, and SY at the 1% and 5% significance level, respectively. Each trait had a positive and statistically significant correlation between SCA and the heterosis levels at the 1% significance level.

**Table 6:** Correlation coefficients between GD, heterosis levels, and combining abilities of each trait.

Trait	GD			SCA		MPH		BPH	
	MPH	BPH	SCA	MPH	BPH	GCA_f_	GCA_m_	GCA_f_	GCA_m_
SY	0.05	0.06	0.19*	0.43**	0.49**	0.36**	0.19*	0.35**	0.27**
SS	0.13	0.08	0.43**	0.34**	0.31**	0.16	−0.09	0.15	−0.07
SP	−0.11	−0.13	0.04	0.55**	0.53**	0.37**	0.18*	0.34**	0.27**
TSW	−0.12	−0.32**	−0.35**	0.54**	0.70**	0.34**	0.05	0.59**	0.11
OC	−0.01	0.08	−0.08	0.53**	0.54**	0.05	−0.04	−0.17*	−0.12

GD: genetic distance; GCA_f_: GCA of female parents; GCA_m_: GCA of male parents.

* Significant at p = 0.05; ** significant at p = 0.01.

The relationships between heterosis level and GCA of the maternal or paternal parent were computed (Table 6). OC and TSW showed statistically significant correlations between heterosis level and GCA of maternal parents at the 5% and 1% significance level, respectively. SP and SY displayed positive and statistically significant correlations between heterosis levels and GCA of both parents at the 5% or 1% significance level. SS showed no significant correlation between heterosis levels and GCA of both parents.

### Performances of hybrids are closely related to parents

**Figure 1:**
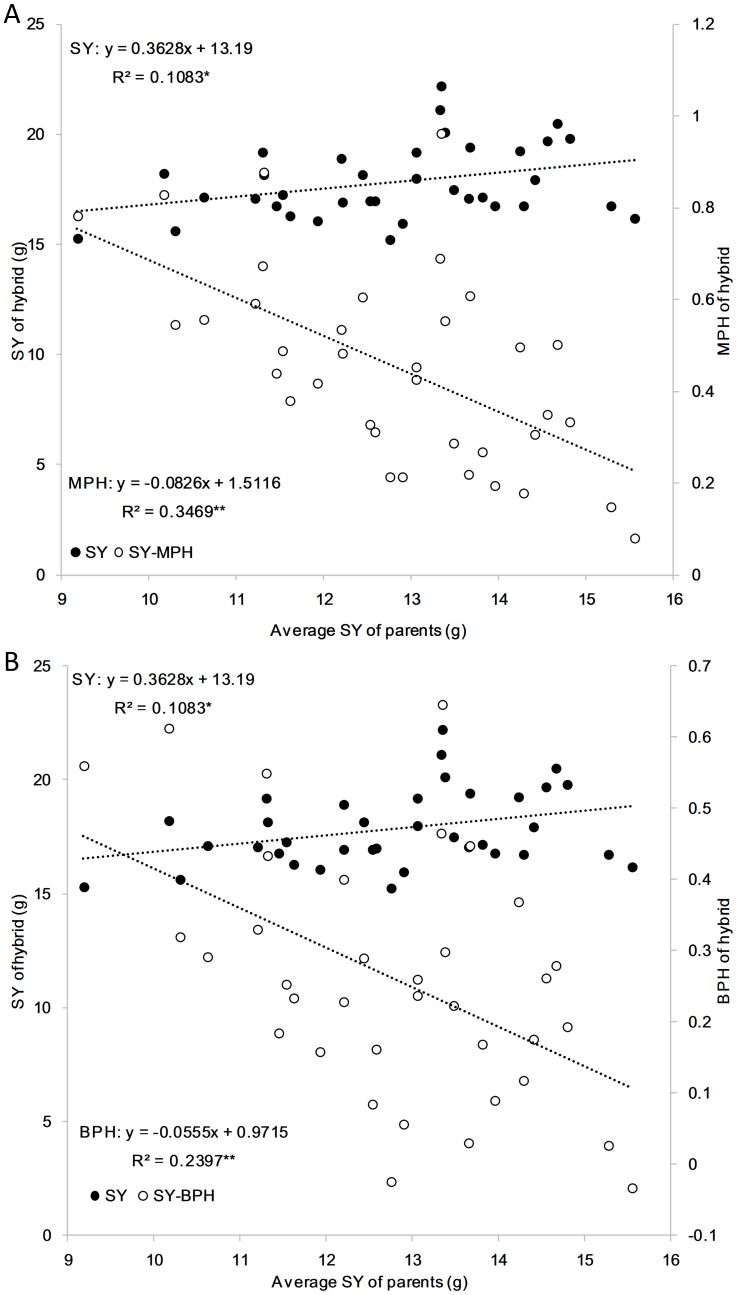
Relationships between average performances of parents and their hybrids for heterosis levels of seed yield. (A) relationship with MPH. (B) relationship with BPH. * Significant at p = 0.05; ** significant at p = 0.01; n = 36

Correlation coefficients between performance of hybrids and their parents were found to be positive and statistically significant at the 1% significance level (Table 7). In addition, the correlations between performance of hybrids and the average performance of the two parents were also statistically significant at the 1% significance level. However, the correlations between heterosis levels and the average performance of their parents were negative and statistically significant at the 1% or 5% significance level, except for TSW (Figure 1, Table 8).

**Table 7:** Correlation coefficients of each trait between hybrid and its parental lines.

Trait	Hybrid and female parent	Hybrid and male parent	Hybrids and two parents
SY	0.69**	0.65**	0.76**
SS	0.53**	0.62**	0.74**
SP	0.60**	0.77**	0.75**
TSW	0.76**	0.50**	0.81**
OC	0.55**	0.65**	0.77**

** Significant at p = 0.01.

**Table 8:** Correlation coefficients between heterosis level and average performance of parents.

Heterosis level	Traits				
	SY	SS	SP	TSW	OC
MPH	−0.43**	−0.52**	−0.35**	−0.16	−0.46**
BPH	−0.35**	−0.30**	−0.32**	0.09	−0.42**

** Significant at p = 0.01; n = 36.

Correlations of phenotypes among the 5 traits were computed with parents as the homozygous genotype and hybrids as the heterozygous genotype, respectively (Table 9). Positive and statistically significant correlations were detected between SY and SP, and between SS and OC at the 1% significance level in both homozygous and heterozygous genotypes, while a negative correlation were found between SS and TSW. However, negative and statistically significant correlations were detected only in heterozygous genotypes between SY and OC, and between SP and two other traits (SS and OC) at the 5% and 1% significance level, respectively.

**Table 9:** Phenotypic correlations between traits in hybrids and parents.

Trait	SY	SS	SP	TSW	OC
SY		−0.12	0.75**	0.11	−0.18*
SS	0.16		−0.41**	−0.60**	0.36**
SP	0.73**	−0.14		−0.03	−0.44**
TSW	0.07	−0.66**	−0.08		0.06
OC	−0.00	0.47**	−0.24	−0.06	

The numbers in the upper or lower triangle are correlation coefficients between traits in hybrids, or between traits in parental lines.

* Significant at p = 0.05; ** significant at p = 0.01; n = 144.

## Discussion

In this study, all 5 traits appeared to have high heterosis levels and a wide variations (Table 4), which is consistent with previous studies [12]–[13]. This result suggests great potential for improving yield-related traits in rapeseed through hybrid breeding.

Though heterosis of rapeseed appears to be strong, it has not been utilized fully in hybrid breeding. One of the reasons is the difficulty in predicting heterosis level from parental generation [1], [14]. To explore an effective way to heterosis prediction, relationships of GD with combining abilities and heterosis levels were extensively studied, and significant correlations were detected in some traits in the previous studies [1], [8], [10], [34]. However, contradictory results for correlations between GD and heterosis of seed yield and some agronomic traits were also reported [10]–[11]. In our study, GD was significantly correlated only with BPH of TSW, and SCA of SY, TSW, and SS (Table 6), which suggested that GD is a valuable genetic parameter for predicting heterosis levels based on the molecular markers [27], [35], but should be used with cautions and with other parameters together.

Another way to hybrid prediction is to use the estimates of combining ability. Qian et al. and Rameeh found significant correlations between heterosis levels and combining abilities in rapeseed [13], [36]. However, no significant correlation was found between GD and heterosis levels in prior research [13]. In maize, significant correlations were found between heterosis levels and combining abilities [15], [37]. Similarly, our study found significantly positive correlations between combining abilities and heterosis levels in all 5 traits except for GCA of SS. Our results together with previous studies indicate that combining ability may be considered a more effective parameter than GD in heterosis prediction of rapeseed.

Thus far, the contributions of GCA variance of different parents together with maternal and paternal effects to hybrid performance have been rarely studied, especially in rapeseed [24]. In our study, the contribution ratios of the two parental GCA variances as well as the correlations between heterosis levels and GCA of parents showed that TSW was mainly controlled by the maternal genotype, and the maternal effect was 55.22% higher than paternal effect. Furthermore, our study showed that heterosis levels had a statistically significant and positive correlation with GCA of maternal parent (Tables 5 and 6). SS was also controlled by maternal genotype, while for OC, the contribution of maternal parent was slightly higher than that of paternal parent, which was different from Wang et al. [38]. The maternal effect of various seed traits including seed size was shown in many other crops to be correlated with parental environmental effects (e.g. availability of light, temperature, water, and nutrients) and cytoplasmic effects [39]–[40]. Based on the contribution ratio of GCA variance and correlation between heterosis levels and GCA of parents, paternal parent contributed more than maternal parent for SY, which provided valuable information for SY improvement in hybrid breeding of rapeseed. For SP, paternal parent contributed slightly more than maternal parent. Higher paternal effect on inheritance was also found in the leaflet area and other traits in carrot [41]. This paternal effect is likely contributed by epigenetic mechanisms [42], and plays an important role in the expression of polyphenic traits [43].

To the best of our knowledge, no report has to date has explored the relationships between traits in parental lines and their hybrids of rapeseed. Our study revealed that the correlations between SS and TSW, SS and OC, and SY and SP are statistically significant in both parents and hybrids (Table 9). In contrast, correlations between SS and SP, SP and OC, and SY and OC only appeared to be statistically significant in hybrids. The relationships between traits that are different from those obtained from homozygous genotypes or homozygous genotypes mixed with heterozygous genotypes provide valuable information for hybrid breeding.

Previously, additive, dominant, and epistasis effects were all detected for yield and yield related traits in rapeseed hybrids [19]. Zhang et al. found that SS was mainly controlled by the additive effect [44], which was similar to the results from our study (Table 2, and 5). In contrast to the result of Amiri-Oghan et al. [17], we found SY was mainly controlled by the additive by additive effect (Table 3). However, dominant effect was also detected for SY inheritance by Shen et al. and Radoev et al. [45]–[46]. Interestingly, traits primarily controlled by maternal parent were mainly additive inheritance, and traits primarily controlled by paternal parents were mainly dominant inheritance in our study. The reason for this phenomenon is unknown, and further studies are needed to understand the mechanism underlying this phenomenon.

In conclusion, hybrid performance in hybrid breeding might be predicted by combining ability supplemented with GD. Furthermore, the selection of maternal and paternal parents should be based on the maternal or paternal control of traits in hybrid breeding. For example, parents with a higher combining ability of TSW should be selected as a female parent, while parents with a higher combining ability of SY as a male parent. Our study provides useful information for parental selection in hybrid breeding of rapeseed and other crops.

## Supporting Information

Table S1 Major characteristics of the parental lines used for incomplete diallel crosses.
(DOC)
