# Supplementary material for: Parental Selection of Hybrid Breeding Based On Maternal and Paternal Inheritance of Traits in Rapeseed (Brassica napus L.)
Source: PLoS One. 2014 Jul 25;9(7):e103165. doi: 10.1371/journal.pone.0103165 (PMC4111582; doi:10.1371/journal.pone.0103165)
Supplement: Table S1 — Major characteristics of the parental lines used for incomplete diallel crosses. (DOC) [file pone.0103165.s001.doc]

**Table S1 Major characteristics of the parental lines used for incomplete diallel crosses**

| Code | Identity | Source | Growth period  (days)^1^ | Yield level  (g/plant)^2^ | Oil content  (%)^2^ | Quality^3^ |
| --- | --- | --- | --- | --- | --- | --- |
| P1 | Zhongshuang 5 | Registered cultivar | 217 | 15.71 | 40.73 | Double low |
| P2 | Zhongshuang 7 | Registered cultivar | 225 | 13.93 | 40.23 | Double low |
| P3 | Zhongshuang 10 | Registered cultivar | 226 | 11.65 | 44.85 | Double low |
| P4 | JiaA177 | Pure line | 228 | 14.20 | 39.89 | Double low |
| P5 | Jia5754 | Pure line | 230 | 9.66 | 39.37 | Double low |
| P6 | Jia5551 | Pure line | 219 | 15.16 | 40.63 | Double low |
| P7 | Zhongshuang 9 | Registered cultivar | 229 | 11.58 | 40.33 | Double low |
| P8 | Huashuang 5 | Registered cultivar | 214 | 15.40 | 41.43 | Double low |
| P9 | Jia5739 | Pure line | 225 | 13.40 | 44.42 | Double low |
| P10 | Jia5088 | Pure line | 226 | 10.94 | 38.73 | Double low |
| P11 | Jia5628 | Pure line | 219 | 12.75 | 39.69 | Double low |
| P12 | Jia5657 | Pure line | 225 | 8.70 | 39.10 | Double low |

^1^ The growth period was based on the observation at Wuhan in the season of 2010-2011.

^2^ The yield level and oil content were the average of 4 growth environments.

^3^ Double low means that the content of erucic acid and total glucosinolates in seeds are below 2% and 30 micro mol/g seeds, respectively.
